# Construction of EpCAM overexpression and knockdown vectors and their implications in colorectal cancer research

**DOI:** 10.3389/fgeed.2025.1679698

**Published:** 2025-11-20

**Authors:** Bingping Wang, Jinkai Duan, Jie Zhou, Hulin Ma, Meng Ren, Liangquan Chen, Rina Su, Hao Zhang, Shuang Zhang, Yanwei Gao

**Affiliations:** 1 Department of Abdomina Surgical Oncology, Inner Mongolia People’s Hospital, Hohhot, China; 2 Department of Public Education, Inner Mongolia Technical College of Construction, Hohhot, China; 3 Chilechuan Dairy Development Zone, Inner Mongolia Medical University, Hohhot, China

**Keywords:** epithelial cell adhesion molecule, CRISPR/Cas9, colorectal cancer, translational models, metastasis

## Abstract

**Background:**

The functional characterization of Epithelial Cell Adhesion Molecule (EpCAM) in colorectal cancer (CRC) progression has been constrained by methodological limitations, particularly the potential for truncated protein isoforms to confound traditional genetic knockout approaches. This study aimed to develop a novel CRISPR/Cas9 strategy to overcome this challenge and systematically elucidate the context-dependent oncogenic roles of EpCAM across diverse CRC models.

**Methods:**

We engineered EpCAM overexpression (pCDH-EpCAM) and CRISPR/Cas9 knockdown (pGMC-KO-EpCAM) vectors using restriction digestion and T4 DNA ligation. A strategic dual-exon targeting approach (exons 1 and 3) was employed to minimize the risk of functional escape variants. Selected CRC cell lines (HT-29, HT-115, HRT-18) were genetically modified using optimized Lipofectamine 2000 transfection. Functional impacts were quantitatively assessed through: (i) flow cytometry for EpCAM surface expression (CD326-PE); (ii) daily cell counting over 8 days for proliferation kinetics; and (iii) scratch wound healing (0/24/48 h) and Transwell migration assays (8-μm pores, 18 h) to evaluate metastatic potential.

**Results:**

Successful genetic modulation was achieved and validated: HT-29-OE-EpCAM-2 exhibited an 89% EpCAM-positive rate versus 12% in wild-type (WT) (*p*<0.001), while HRT-18-KD-EpCAM-3 showed a significant reduction to 4% EpCAM-positive cells (vs. 15% in WT, *p*<0.001). EpCAM overexpression accelerated proliferation, with HT-29-OE cells showing a 20.1% increase in peak density on day 5 (30.76 ± 0.15 × 10^4^ vs. WT 25.62 ± 0.25 × 10^4^; *p*<0.001). Conversely, EpCAM knockdown in HRT-18 cells prolonged the doubling time by 8.8% (30.8 h vs. WT 28.3 h; *p*<0.05). Migration capacity was profoundly altered: HT-115-OE cells achieved complete scratch closure (100% vs. 74.05% in WT, *p*<0.001), whereas HRT-18-KD cells showed an 80.5% reduction (*p*<0.001). Transwell migration hierarchy confirmed the pro-metastatic role of EpCAM (HT-29-OE > HT-115-OE > HRT-18-KD; ANOVA *p* = 0.0024).

**Conclusion:**

This study establishes a robust dual-vector toolkit for reliable EpCAM manipulation, highlighting a novel exon-targeting strategy that mitigates the limitations of previous approaches. Our findings demonstrate that EpCAM is a master regulator of CRC aggressiveness, dictating proliferative and metastatic phenotypes in a cell context-dependent manner. The genetically defined models provide a validated platform for therapeutic screening and safety assessment, forming a foundational resource for advancing EpCAM-targeted therapies and diagnostic applications.

## 1 Introduction

Colorectal cancer (CRC) is one of the most prevalent and lethal malignant tumors globally, ranking third in incidence and second in mortality among all cancers. In China, the incidence and mortality rates of CRC remain high, closely interconnected with the interactions between genetic and environmental factors. The development and progression of CRC involve significant genomic alterations, leading to elevated expression levels of specific genes while others may be downregulated. By investigating and validating the causes of these gene expression differences, we can achieve a better understanding of the molecular mechanisms underlying tumorigenesis, ultimately aiding in the identification of effective strategies for early diagnosis and treatment.

Epithelial Cell Adhesion Molecule (EpCAM), a transmembrane glycoprotein encoded by the GA-733–2 gene and approximately 40 kDa in size, is a homophilic, calcium-independent cell adhesion molecule. EpCAM plays a pivotal role in the process of epithelial carcinogenesis, with research indicating that it is crucial for tumor cell adhesion, proliferation, migration, and epithelial-mesenchymal transition (Ezenkwa et al., 2023; Lee et al., 2024). Furthermore, EpCAM is capable of enriching, identifying, and characterizing metastatic cells that spread from primary tumors into the fluids of patients with advanced cancer (Treitschke et al., 2023; Mederer et al., 2022).

The exact molecular mechanisms by which the EpCAM gene influences cellular adhesion remain contentious. Some studies suggest that overexpression of EpCAM may disrupt the interactions among E-cadherin, α-catenin, and F-actin, potentially impairing functional cell adhesion and reducing overall adhesive strength (Balzar et al., 1998; Winter et al., 2003). Conversely, other studies indicate that EpCAM knockout can compromise tissue integrity by lowering surface E-cadherin levels while increasing the levels of tight junction protein 1 (Tjp1) (Slanchev et al., 2009). Moreover, it has been suggested that the functional role of EpCAM in adhesion does not conform to the characteristics of traditional homophilic adhesion molecules, as regulation of EpCAM through intracellular proteolysis and its knockdown has shown little measurable impact on cell-matrix and cell-cell adhesion in cancer cell lines (Tsaktanis et al., 2016; Gaber et al., 2020). Thus, the precise molecular functions of EpCAM in adhesion demands further exploration.

Notably, EpCAM exhibits high expression levels in various cancers, tumor-initiating cells, and circulating tumor cells (Keller et al., 2019; Menyailo et al., 2021; Agnoletto et al., 2021), and is therefore considered a promising therapeutic target for cancer treatment. Various monoclonal antibodies, targeted drugs, and selective antibodies that target EpCAM have shown effectiveness in treating gastrointestinal tumors, metastatic colorectal cancer, prostate cancer, and pancreatic cancer (Hosono et al., 2020; Macdonald et al., 2018; Jiang et al., 2023; Mirzaei et al., 2023; Xu et al., 2023). However, the expression of EpCAM in healthy epithelial cells has led to clinical implications such as gastrointestinal toxicity, pancreatitis, and tolerance issues, resulting in the discontinuation of numerous clinical trials (Gires et al., 2020; Patriarca et al., 2012; Schmidt et al., 2012).

A critical challenge in CRISPR/Cas9-mediated EpCAM knockout is the potential for alternative splicing events that bypass the targeted exon, leading to truncated but functionally competent isoforms. Conventional strategies often target exon 2, which may result in in-frame splicing between exons 1 and 3, producing a residual functional protein (Bagheri et al., 2022). To overcome this limitation, we pioneered a novel strategy by simultaneously targeting exons 1 and 3. This approach is designed to disrupt both the initiation codon and the core structural domain of EpCAM, theoretically preventing the generation of any functional escape variants. This dual-exon targeting represents a significant methodological advancement in EpCAM genetic engineering.

In conclusion, the EpCAM gene plays a vital role in tumor development and progression, and its elevated expression in tumors makes it a potential therapeutic target. Nevertheless, the expression of EpCAM in normal epithelial cells complicates its utility in clinical applications. Therefore, this study employs genetic engineering techniques to construct an EpCAM overexpression vector and a CRISPR/Cas9 knockdown vector, while also establishing colorectal cancer cell lines with EpCAM overexpression as well as downregulated or knocked down EpCAM. This study not only provides essential tools for researching the EpCAM gene’s role in colorectal cancer but also establishes a strong foundation for targeted therapy and immunotherapy research.

## 2 Materials and methods

### 2.1 Construction of the EpCAM overexpression vector

To construct the EpCAM overexpression vector, the human EpCAM gene sequence was retrieved from NCBI. During the synthesis of the full-length EpCAM DNA sequence, XbaI and NheI restriction sites were incorporated at both ends. This sequence was then inserted into the pCHD-CMV-MCS-EF1-RFP-T2A-puro empty vector (SBI, Japan) through these restriction sites, resulting in the construction of the pCDH-EpCAM recombinant expression vector. *E. coli* DH5α(Beijing Qian Shi Jin Biotechnology Co., Ltd.) harboring this recombinant vector was cultured overnight on ampicillin-resistant LB plates at 37 °C(Thermo Scientific Forma Series incubator). A single monoclonal colony was selected for plasmid amplification. The extracted plasmid was subjected to restriction digestion with XbaI/NheI (Thermo Scientific FastDigest enzymes) and analyzed by agarose gel electrophoresis (Bio-Rad PowerPac™ Basic power supply and ChemiDoc™ MP Imaging System) to verify correct insertion. The construct with the expected digestion pattern was submitted to Qingke Biotechnology Co., Ltd. for Sanger sequencing. Monoclonal cultures with confirmed correct sequences were expanded, and high-quality plasmid DNA was purified using an endotoxin-free plasmid extraction kit (Omega Bio-Tek, E. Z.N.A.^®^ Endo-Free Plasmid Mini Kit I). The purity and concentration of the plasmid DNA were quantitatively assessed (NanoDrop™ One/OneC Microvolume UV-Vis Spectrophotometer, Thermo Scientific), and the prepared plasmids were stored at −20 °C for subsequent experiments.

### 2.2 Construction of the EpCAM knockdown vector

sgRNA was designed using the CRISPR ERA website, with the U6 promoter serving as the driving promoter (Table 1). During the synthesis of the full-length gene DNA sequence, NotI and EcoRI restriction sites were added at both ends of the sgRNA. This sequence was ligated into the empty pGMC00010 vector (Addgene) using these restriction sites, resulting in the pGMC-KO-EpCAM knockdown vector. *E. coli* DH5α containing this recombinant vector were cultured overnight on ampicillin-resistant LB plates at 37 °C. Selected monoclonal colonies were sent for sequencing, using a primer sequence of F: GTT​CGG​AAA​CCT​GAT​TGC. The monoclonal colonies with confirmed sequences were expanded, and plasmids were extracted using an endotoxin-free extraction kit before storing at −20 °C.

**TABLE 1:** sgRNA sequences for constructing the EpCAM knockdown vector.

Vecror		sgRNA	Position on the EpCAM	Exon
PGMC-KO-EpCAM-1	sgRNA-1	GTT​CGG​GCT​TCT​GCT​TGC​CG	219–238	1
	sgRNA-2	GGC​GAC​TTT​TGC​CGC​AGC​TC	246–265	1
PGMC-KO-EpCAM-2	sgRNA-3	GAT​CCT​GAC​TGC​GAT​GAG​AG	481–500	3
	sgRNA-4	GCA​ACG​GCA​CCT​CCA​TGT​GC	524–543	3
PGMC-KO-EpCAM-3	sgRNA-5	GCT​TCT​GCT​TGC​CGC​GGC​GA	225–244	1
	sgRNA-6	GGG​GCC​CTC​CAG​AAC​AAT​GA	451–470	3

### 2.3 Culture of colorectal cancer cell lines

The culture medium for the HT-29 colorectal cancer cell line consisted of 90% McCoy’s 5A medium (Gibco, United States) and 10% FBS (Gibco, United States); the culture media for HT-115 and HRT-18 were 90% DMEM high glucose (Gibco, United States) and 10% FBS; the T84 cell line was maintained in 95% DMEM/F12 (Gibco, United States) and 5% FBS; LOVO was grown in 90% Ham’s F12k medium (Gibco, United States) and 10% FBS; COLO205 in 90% RPMI1640 (Gibco, United States) and 10% FBS; and CaCO2 in 90% DMEM high glucose (Gibco, United States) and 10% FBS. AII of the colorectal cancer cell lines were acquired from the Shanghai Zhong Qiao Xin Zhou.

To thaw the cells stored in liquid nitrogen, they were quickly placed in a 37 °C water bath while gently shaking to ensure timely thawing within 1 minute. After disinfection, the thawed cells were quickly transferred to a sterile cabinet. The cell suspension was transferred to a 1.5 mL tube, centrifuged at 1,500 rpm for 5 min, and the supernatant was discarded. One mL of fresh culture medium was added to resuspend the cells, and they were counted before being seeded into T25 flasks at a density of 1 × 10^6 cells per flask. The cells were then cultured at 37 °C in a 5% CO2 incubator (Thermo, United States). Once cells reached approximately 90% confluence, they were trypsinized with 0.25% trypsin (Gibco, United States) for about 2 min. COLO205 is a semi-adherent cell line, whereas the other colorectal cancer cells were adherent; thus, care was taken to collect any suspension cells during media changes and passaging of COLO205.

### 2.4 Flow cytometric analysis

Flow cytometric analysis assessed the expression of the EpCAM gene across seven colorectal cancer cell lines and their corresponding transgenic variants. For each cell line, 1 × 10^6 cells were collected, washed twice with PBS (Gibco, United States) to remove culture media and serum, and stained with CD-326-PE antibodies for 30 min. Afterward, two washes with PBS helped eliminate any unbound antibodies, and cells were resuspended in PBS for flow cytometric evaluation (FACSCalibur flow cytometers from BD, United States).

### 2.5 Puromycin cytotoxicity assay

Each colorectal cancer cell line was seeded into 24-well plates at a density of 5 × 10^4 cells per well. After 24 h, the culture medium was replaced with fresh media containing varying concentrations of puromycin (Solarbio, China), with three replicates per concentration. The concentration levels assessed included 0, 1, 2, 3, 4, 5, 6, 7, and 8 ng/mL. Fresh media were replaced every 2 days, and the concentration that led to complete cell death by days 10–14 was defined as the screening concentration for transgenic cells. Half of this concentration was designated as the maintenance concentration for the transgenic cell lines.

### 2.6 Construction of transgenic cells

Transfection conditions were optimized prior to formal experiments. Briefly, cells were seeded in 24-well plates and transfected with a control GFP plasmid using Lipofectamine 2000 (Thermo Fisher Scientific) at varying DNA (μg) to reagent (μL) ratios (1:1, 1:2, 1:3, 1:4). Transfection efficiency was assessed 24–48 h post-transfection by calculating the percentage of GFP-positive cells using fluorescence microscopy (Nikon Eclipse Ti2). The optimal ratio yielding the highest efficiency with minimal cytotoxicity (1:2.5 for HT-29,T84 and HRT-18; 1:3 for HT-115) was selected for all subsequent transfections. To establish transgenic cells, the selected colorectal cancer cell lines were initially seeded into 24-well plates. Once approximately 80% confluence was achieved, the medium was changed to serums-free media and remained for 2 hours before transfection. The transfection reagent Lipo2000 and the target plasmid preparations were each added to opti-MEM (Gibco, United States), mixed after sitting for 5 min, and incubated in the dark for 20 min. The resulting solution was applied to the cell wells. After a 6-h incubation, normal media containing serum replaced the transfection solution, and transfection efficiency was assessed using a fluorescence microscope 18–24 h later. Cells were passaged based on the transfection efficiency at ratios ranging from 1:10 to 1:45 into 10 mm dishes. The appropriate puromycin concentration was added based on earlier cytotoxicity assay results, and fresh selection media were replaced every 3 days. Following 10–14 days, monoclonal cell colonies were observed, and these monoclonal cells were collected using a cloning ring for passaging. Once adequate scalability was reached, flow cytometry was used to confirm each monoclonal cell line, ultimately yielding the necessary transgenic cell lines for this study.

### 2.7 Cell proliferation assay

Cell proliferation was quantified by manual cell counting and growth curve plotting. Briefly, CRC cells (1 × 10^4^ cells/well) were seeded in 24-well plates. Triplicate wells were trypsinized and counted daily for 8 consecutive days using a hemocytometer under phase-contrast microscopy (Olympus CKX53). Growth curves were generated by plotting cell numbers against time. Data normalization was performed against the initial seeding density (Day 0).

### 2.8 Cell migration assay

Scratch wound healing: Confluent monolayers in 24-well plates were scratched with 200 μL pipette tips. Wound closure (%) was measured at 0/48 h using ImageJ v1.53.

Transwell migration: Cell migration ability was assessed using 24-well Transwell chambers with 8.0 μm pore polycarbonate membranes (Corning, United States). Briefly, cells were serum-starved for 12 h prior to the assay. Subsequently, 5 × 10^4^ cells in 200 μL of serum-free medium were seeded into the upper chamber. The lower chamber was filled with 600 μL of complete medium containing 10% FBS as a chemoattractant. After 24 h of incubation at 37 °C in a 5% CO_2_ atmosphere, non-migrated cells on the upper surface of the membrane were carefully removed with a cotton swab. Migrated cells on the lower surface were fixed with 4% paraformaldehyde for 20 min, followed by staining with 0.1% crystal violet for 15 min at room temperature. After washing with PBS to remove excess dye, the membranes were air-dried. Images of five randomly selected fields per membrane were captured using a light microscope (Nikon Eclipse Ti2, Japan) at ×100 magnification. The number of migrated cells was quantified using ImageJ software (v1.53, NIH, United States). Each experiment was performed in triplicate wells and repeated independently at least three times. Data are presented as the mean number of migrated cells per field ±standard deviation (SD).

### 2.9 Statistical analysis

All functional assays (proliferation, scratch wound healing, and Transwell migration) were performed with three independent biological replicates, each containing three technical replicates. Data are presented as the mean ± standard deviation (SD). Statistical comparisons between two groups were analyzed using two-tailed Student’s t-test. A p-value of <0.05 was considered statistically significant. Although a p-value of 0.038 was accepted as statistically significant, results approaching the threshold (p-values between 0.04 and 0.05) are noted and interpreted with caution in the context of the experimental findings. All statistical analyses were performed using GraphPad Prism software (version 9.0).

## 3 Results

### 3.1 Engineering of EpCAM expression vectors

**FIGURE 1:**
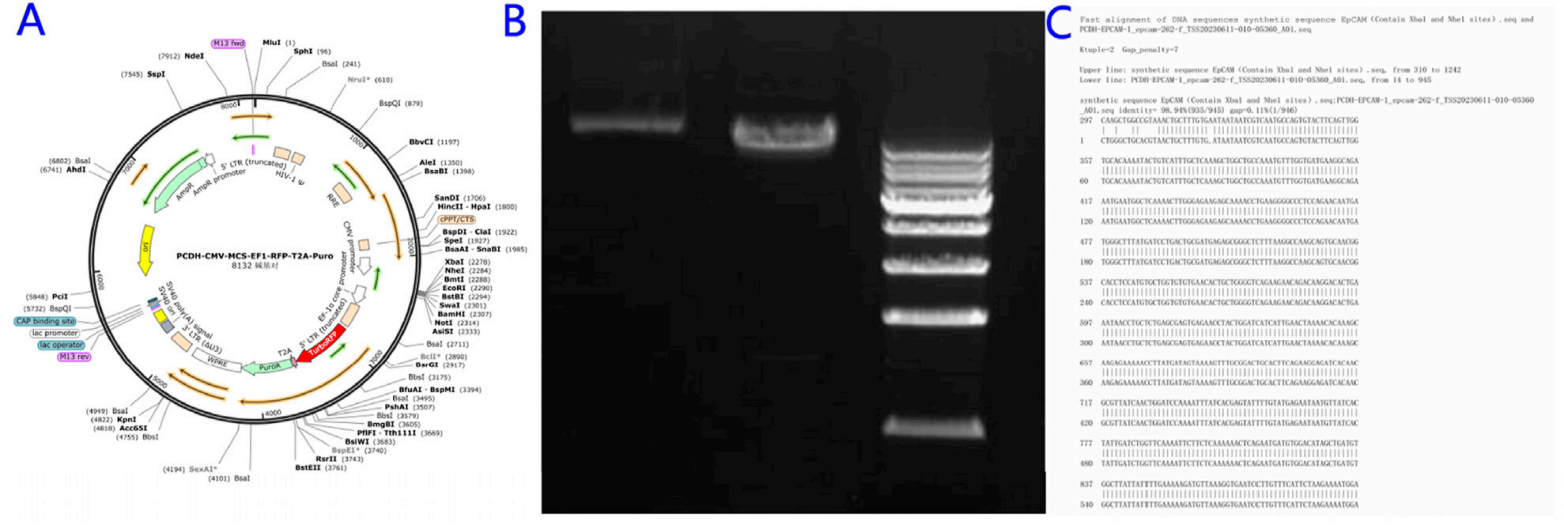
Construction of the EpCAM Overexpression Vector. **(A)** Empty vector map of pCHD-CMV-MCS-EF1-RFP-T2A-puro; **(B)** Mapping map of the recombinant expression vector pCDH-EpCAM; **(C)** Complete consistent sequence alignment of the constructed pCDH-EpCAM recombinant expression vector with the EpCAM gene.

The 1559-bp EpCAM gene fragment was cloned into the pCDH-CMV-MCS-EF1-RFP-T2A-puro backbone (Figure 1A) via XbaI/NheI digestion and T4 DNA ligation. Successful construction of the overexpression vector pCDH-EpCAM was validated by restriction mapping and Sanger sequencing (Figures 1B,C).

### 3.2 CRISPR/Cas9-mediated EpCAM knockdown vector assembly

**FIGURE 2:**
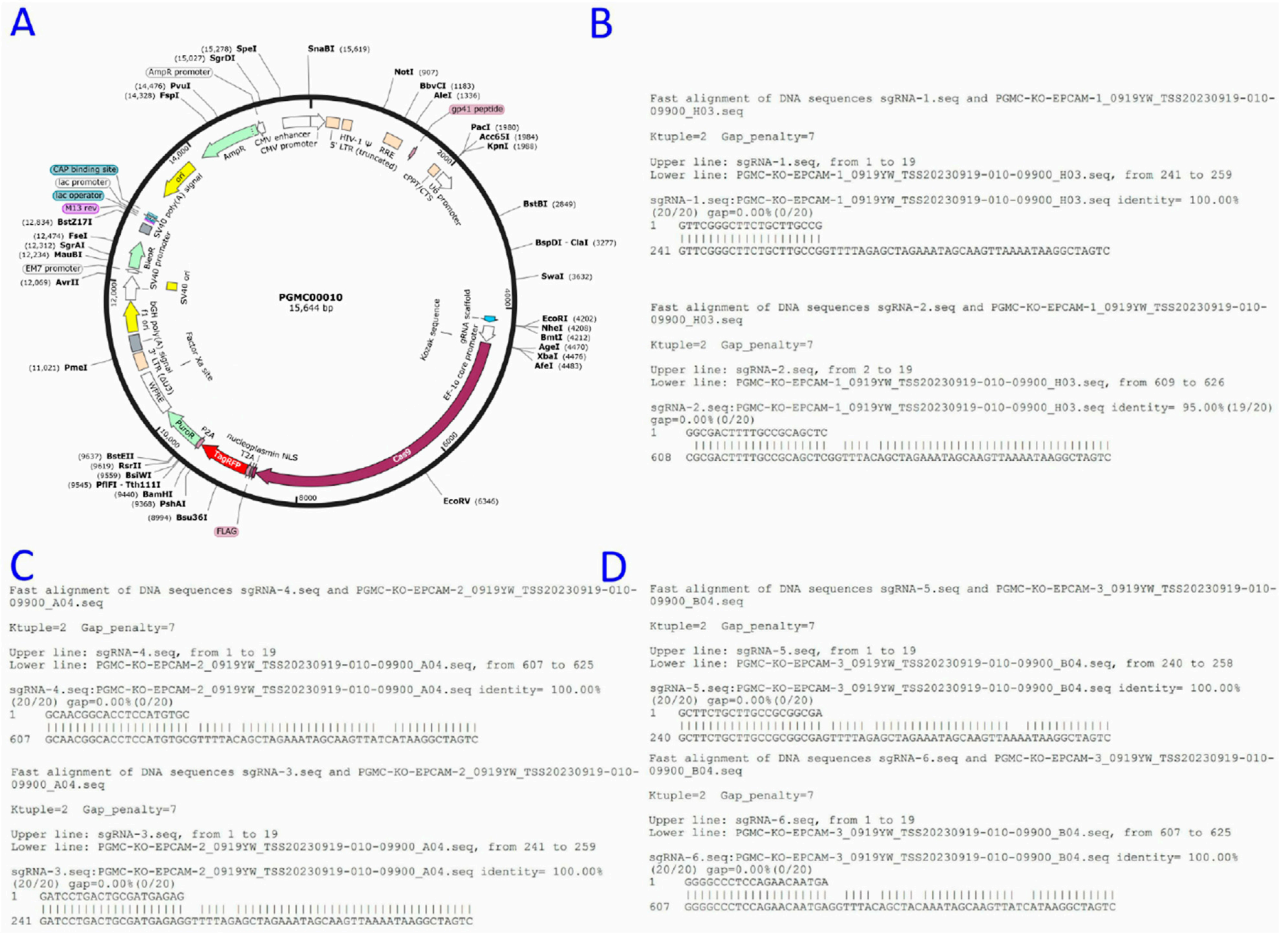
Construction of the EpCAM knockdown Vector. **(A)** Empty vector map of pGMC00010; **(B)** The sequence alignment of the constructed PGMC-KO-EpCAM-1 recombinant expression vector with sgRNA-1 and sgRNA-2 genes, and the results were completely consistent; **(C)** The sequence alignment of the constructed PGMC-KO-EpCAM-2 recombinant expression vector with sgRNA-3 and sgRNA-4 genes, and the results were completely consistent; **(D)**The sequence alignment of the constructed PGMC-KO-EpCAM-3 recombinant expression vector with sgRNA-5 and sgRNA-6 genes, and the results were completely consistent.

Three sgRNAs targeting exon-flanking regions were designed (CRISPR ERA) and ligated into the pGMC00010 vector using NotI/EcoRI sites (Figure 2A). Sequencing confirmed successful generation of knockdown constructs pGMC-KO-EpCAM-1/2/3 (Figures 2B–D).

### 3.3 Heterogeneous EpCAM expression in CRC cell lines

**FIGURE 3:**
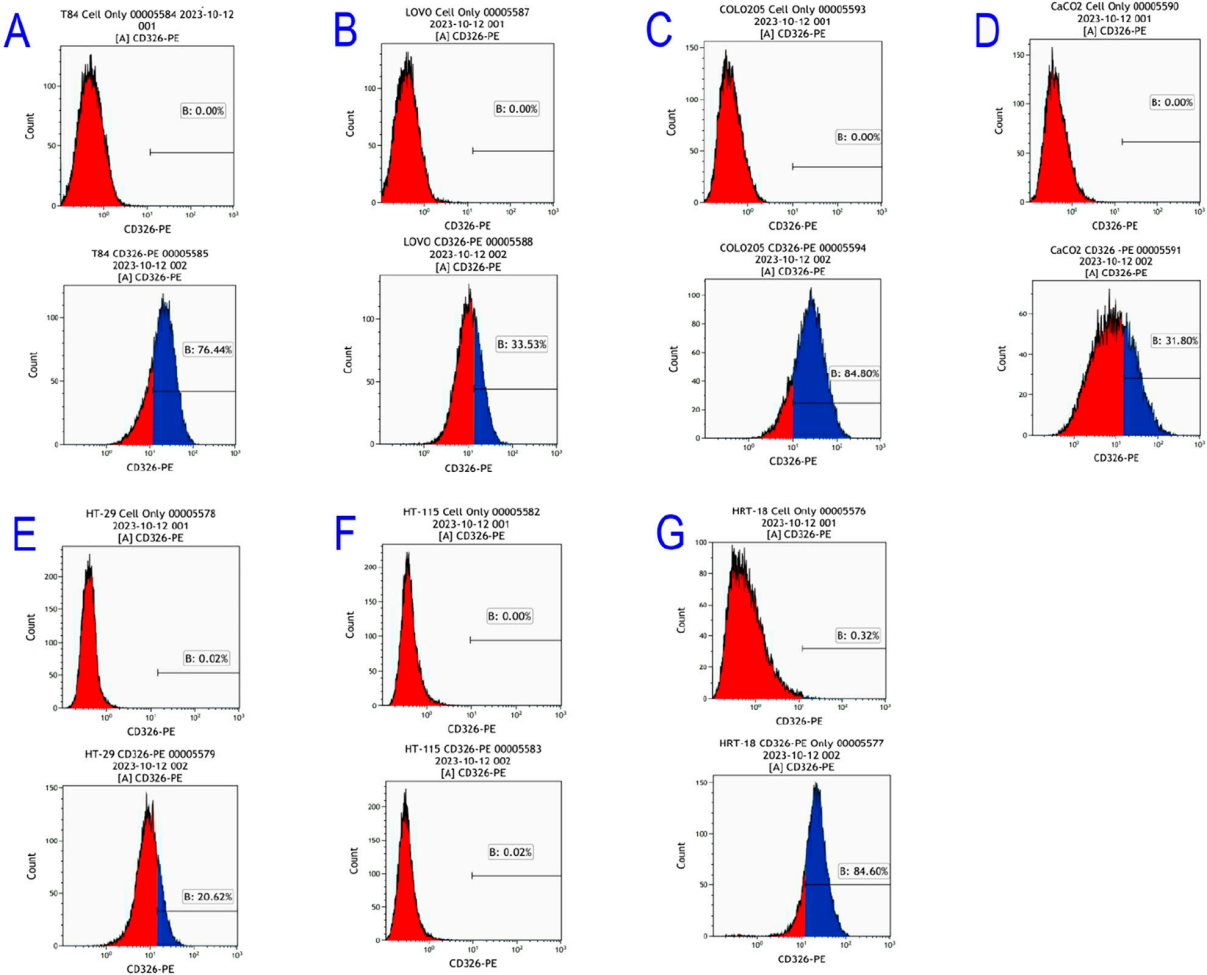
Flow cytometric results of EpCAM gene expression in colorectal cancer cell Lines. **(A)** T84; **(B)** LOVO; **(C)** COLO205; **(D)** CaCO2; **(E)** HT-29; **(F)** HT-115; **(G)** HRT-18.

Flow cytometry revealed differential EpCAM expression across seven CRC lines (Figures 3A–G):

High expressors: COLO205 (84.8%), T84 (76.4%), HRT-18 (84.6%)

Moderate expressors: LOVO (33.53%), CaCO2 (31.8%), HT-29 (20.62%)

Non-expressors: HT-115 (0%)

### 3.4 Determination of puromycin selection thresholds

Cell line-specific lethal puromycin concentrations were established via cytotoxicity assays (Table 2), enabling optimal selection pressure during transgenic cell screening.

**TABLE 2:** Puromycin screening concentrations for colorectal cancer cell lines.

Cell line	T84	LOVO	COLO205	CaCO2	HT-29	HT-115	HRT-18
Puromycin concentration (ng/ul)	6	5	4	8	4	6	6

### 3.5 Establishment of EpCAM-Overexpressing CRC lines

**FIGURE 4:**
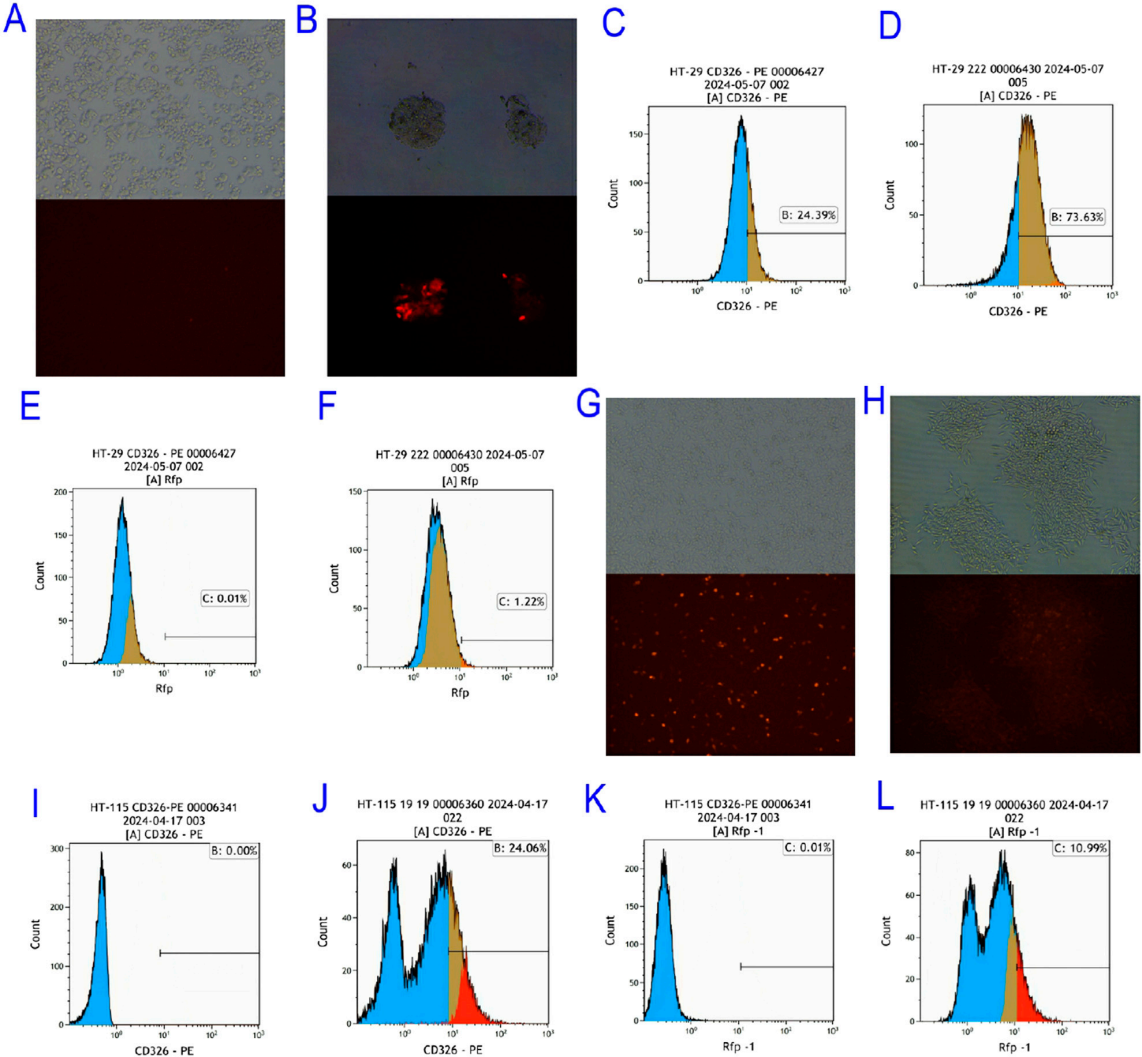
Screening of EpCAM Overexpressing Colorectal Cancer Cell Lines. **(A)** Bright field and red fluorescence excitation 24 h after HT-pCDH-EpCAM overexpression vector of HT-29 colorectal cancer cell line; **(B)** Bright field fields and red fluorescence excitation fields of single clones selected after the HT-29 colorectal cancer cell line was transfected with pCDH-EpCAM overexpression vector; **(C)** The expression of EpCAM gene in HT 29 was 24.39%; **(D)** The expression of EpCAM gene in HT-29-OE-EpCAM-2 transgenic colorectal cancer cell line was 73.63%; **(E)** The expression of RFP gene in HT 29 colorectal cancer cell line was 0.01%; **(F)** The expression of RFP gene in HT-29-OE-EpCAM-2 transgenic colorectal cancer cell line was 1.22%; **(G)** Bright field and red fluorescence excitation field observed 24 h after transfection with pCDH-EpCAM overexpression vector in HT-115 colorectal cancer cell line; **(H)** Bright field and red fluorescence excitation field of the single clone selected from HT-115 after transfection with pCDH-EpCAM overexpression vector of HT-115 colorectal cancer cell line; **(I)** The expression of EpCAM gene in HT 115 colorectal cancer cell line was 0.00%; **(J)** The expression of EpCAM gene in HT-115-OE-EpCAM-19 transgenic colorectal cancer cell line was 24.06%; **(K)** The expression of RFP gene in HT 115 colorectal cancer cell line was 0.01%; L: The expression of RFP gene in HT-115-OE-EpCAM-19 transgenic colorectal-cancer cell line was 10.99%.

The pCDH-EpCAM overexpression vector was transfected into HT-29 and HT-115 cells using Lipofectamine 2000. Successful transfection was confirmed at 24 h by the presence of red fluorescence (RFP) under microscopy (Figures 4A–G). Following puromycin selection (10–14 days), monoclonal expansion was performed. Single clones exhibiting strong RFP fluorescence were isolated and expanded (Figures 4B–H).

The success of EpCAM overexpression was rigorously quantified by flow cytometry. In the HT-29 background, flow cytometry analysis revealed that the transgenic clone HT-29-OE-EpCAM-2 exhibited a significant 3.6-fold increase in EpCAM surface expression compared to wild-type cells (73.63% vs. 20.39% positive cells, respectively; Figures 4C,D). Concurrently, RFP expression served as a transfection efficiency marker, increasing from 0.01% in wild-type to 1.22% in the transgenic clone (Figures 4E,F).

Similarly, in the originally EpCAM-negative HT-115 cell line (0.00% positive; Figure 4I), the engineered clone HT-115-OE-EpCAM-19 successfully achieved EpCAM positivity, with 24.06% of cells expressing EpCAM (Figure 4J). The high RFP expression in this clone (10.99%, Figure 4L) compared to the background (0.01%, Figure 4K) further confirmed the stable integration and expression of the vector.

These results collectively demonstrate the successful establishment of two novel EpCAM-overexpressing CRC cell models with varying basal levels of EpCAM, providing essential tools for subsequent functional studies.

### 3.6 Generation of EpCAM-knockdown cell models

**FIGURE 5:**
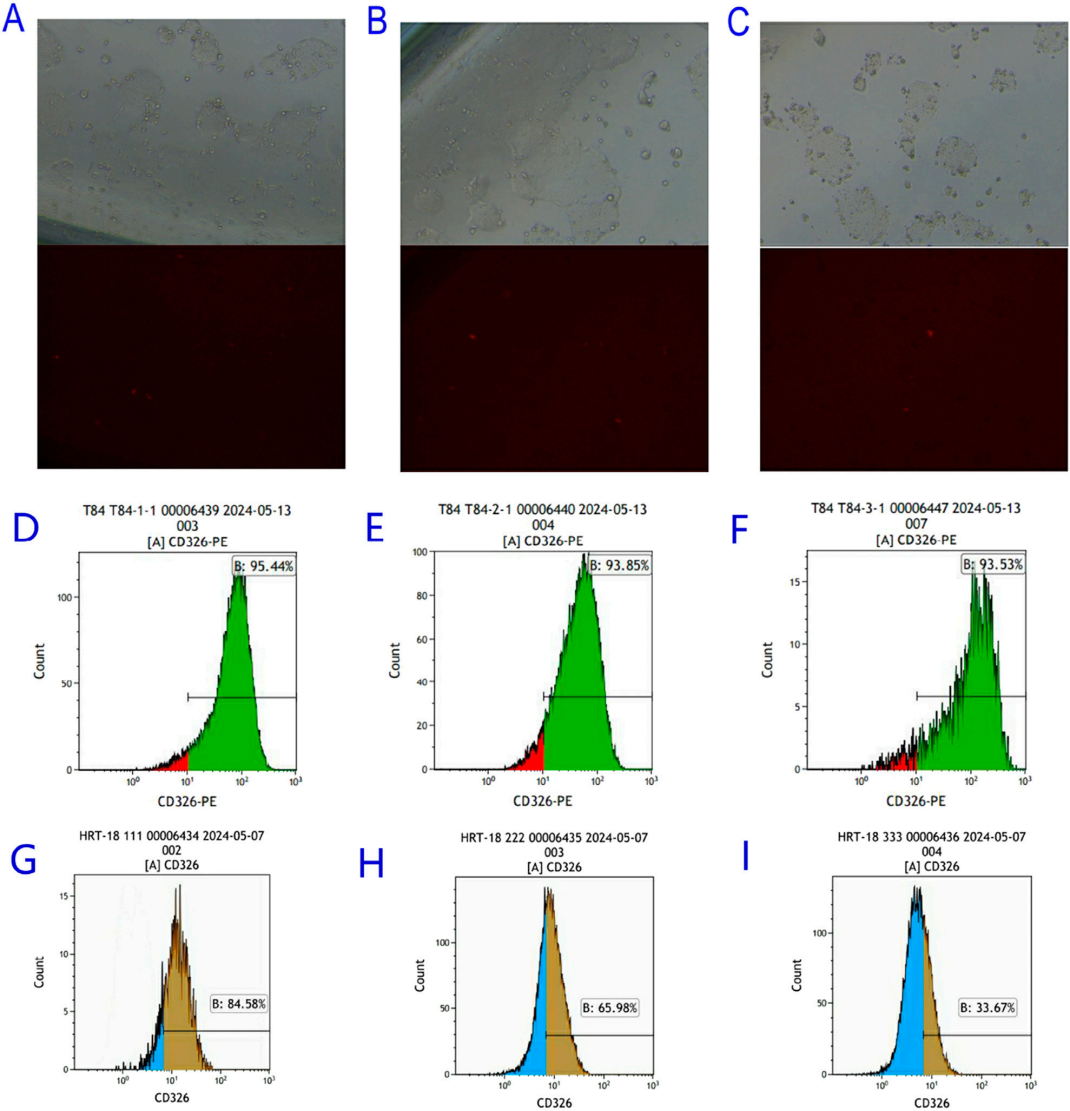
Screening of EpCAM knockdown Colorectal Cancer Cell Lines. **(A)** Bright field and red fluorescence excitation field observed 24 h after transfection of PGMC-KO-EpCAM-1 knockdown vector in T84 colorectal cancer cell line; **(B)** Bright field and red fluorescence excitation 24 h after transfection of PGMC-KO-EpCAM-2 knockdown vector in T84 colorectal cancer 2 cell line; **(C)** Bright field and red fluorescence excitation field observed 24 h after transfection of PGMC-KO-EpCAM-3 knockdown vector in T84 colorectal cancer cell line; **(D)** The expression of T84-KO-EpCAM-1 transgenic colorectal cancer cell line EpCAM gene was 95.44%; **(E)** The expression of T84-KO-EpCAM-2 transgenic colorectal cancer cell line EpCAM gene was 93.85%; **(F)** The expression of T84-KO-EpCAM-3 transgenic colorectal cancer cell line EpCAM gene was 93.53%; **(G)** The expression of HRT-18-KO-EpCAM-1 transgenic colorectal cancer cell line EpCAM gene was 84.58%; **(H)** The expression of EpCAM gene in HRT-18-KO-EpCAM-2 transgenic colorectal cancer cell line was 65.98%; **(I)** The expression of EpCAM gene in HRT-18-KO-EpCAM-3 transgenic colorectal cancer cell line was 33.67%.

pGMC-KO-EpCAM vectors were transfected into HRT-18 and T84 cells. Among the T84 monoclonal cell lines screened, although weak red fluorescence was observed under fluorescence microscopy, flow cytometric analysis confirmed that EpCAM expression was neither knocked out nor knocked down. In contrast, drug screening in HRT-18 cells yielded only two monoclonal lines. Despite the absence of red fluorescence observed under fluorescence microscopy, flow cytometry identified a significant reduction in EpCAM expression, with HRT-18-KO-EpCAM-3 exhibiting 60% EpCAM↓ (flow cytometry, Figure 5).

### 3.7 EpCAM modulates proliferation kinetics in a cell context-dependent manner

To systematically investigate the context-dependent role of EpCAM, we employed three genetically engineered CRC models representing distinct biological backgrounds:

HT-29-OE-EpCAM-2: An isogenic model derived from HT-29 (a cell line established from a primary colon adenocarcinoma metastasis). This model features EpCAM overexpression in a cell line with intermediate endogenous EpCAM expression and inherent metastatic propensity.

HT-115-OE-EpCAM-19: A model derived from HT-115 (a cell line from a primary colon carcinoma with low malignant potential). This model introduces EpCAM overexpression into a cell line that is basically EpCAM-negative, allowing us to test the sufficiency of EpCAM in driving oncogenic transformation.

HRT-18-KO-EpCAM-3: A model derived from HRT-18 (a highly aggressive cell line from a primary colon adenocarcinoma). This model features CRISPR/Cas9-mediated EpCAM knockdown in a cell line with very high endogenous EpCAM expression, enabling us to test the necessity of EpCAM for maintaining malignant phenotypes.

Proliferation profiling across these models demonstrated distinct kinetic alterations:

In the metastatic background of HT-29-OE-EpCAM-2, EpCAM overexpression accelerated exponential growth, yielding a 20.1% increase in maximal cell density (day 5: 30.76 ± 0.15 × 10^4^ vs. WT 25.62 ± 0.25 × 10^4^; *p* < 0.001) and a reduced doubling time (22.4 h vs. 26.1 h, *p* < 0.01).

In the low-malignancy background of HT-115-OE-EpCAM-19, EpCAM overexpression induced an earlier plateau onset (day 5: 25.82 ± 0.12 × 10^4^ vs. WT 23.15 ± 0.46 × 10^4^; *p* = 0.005) accompanied by an 11.5% increase in saturation density.

In the aggressive background of HRT-18-KO-EpCAM-3, EpCAM knockdown resulted in a prolonged log phase (days 3–6), reduced cell counts at day 6 (19.99 ± 0.55 × 10^4^ vs. WT 20.72 ± 0.29 × 10^4^; *p* = 0.038), and an increased doubling time (30.8 h vs. 28.3 h; *p* < 0.05).

**FIGURE 6:**
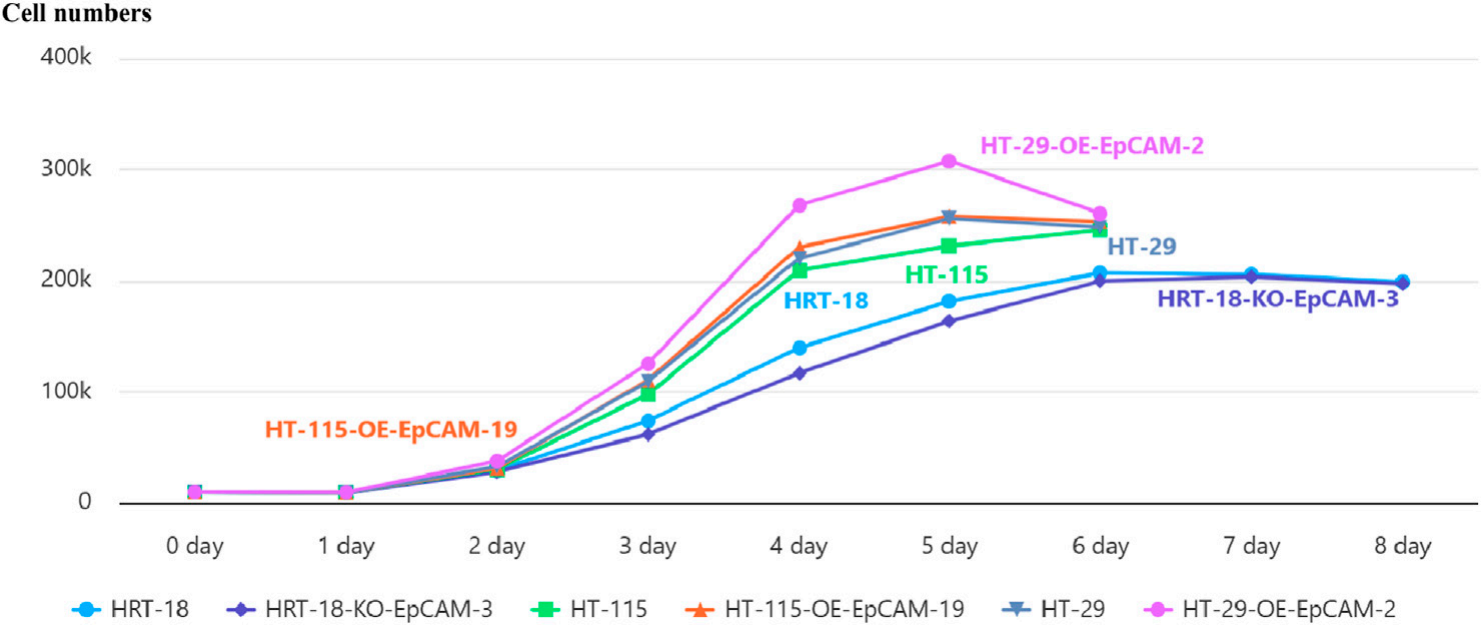
Cell growth curve.

These findings establish that EpCAM universally promotes proliferation across diverse CRC contexts, with the most pronounced effect observed in the metastatic HT-29-OE-EpCAM-2 model (Figure 6; Table 3).

**TABLE 3:** Proliferation kinetics reveal cell line-specific EpCAM functions.

Cell line	Day 5 (×10^4^)	Change vs. WT	Doubling time (h)
HT-29 WT	25.62 ± 0.25	Reference	26.1
HT-29-OE-EpCAM-2	30.76 ± 0.15	↑20.1%***	22.4**
HT-115 WT	23.15 ± 0.46	Reference	27.8
HT-115-OE-EpCAM-19	25.82 ± 0.12	↑11.5%**	25.1*
HRT-18 WT	18.15 ± 0.37	Reference	28.3
HRT-18-KO-EpCAM-3	16.39 ± 0.28	↓9.7%*	30.8*

### 3.8 EpCAM governs migration capacity

#### 3.8.1 Scratch wound healing

HT-29-OE-EpCAM-2: 2.6-fold↑ closure rate (14.08% ± 9.15% vs. WT 5.37% ± 3.80%; *p* = 0.023).

HT-115-OE-EpCAM-19: Complete monolayer repair (100.00% ± 0.00% vs. WT 74.05% ± 3.58%; *p* < 0.001).

HRT-18-KO-EpCAM-3: Severely impaired migration (3.79% ± 3.36% vs. WT 19.45% ± 1.59%; *p* < 0.001).

**FIGURE 7:**
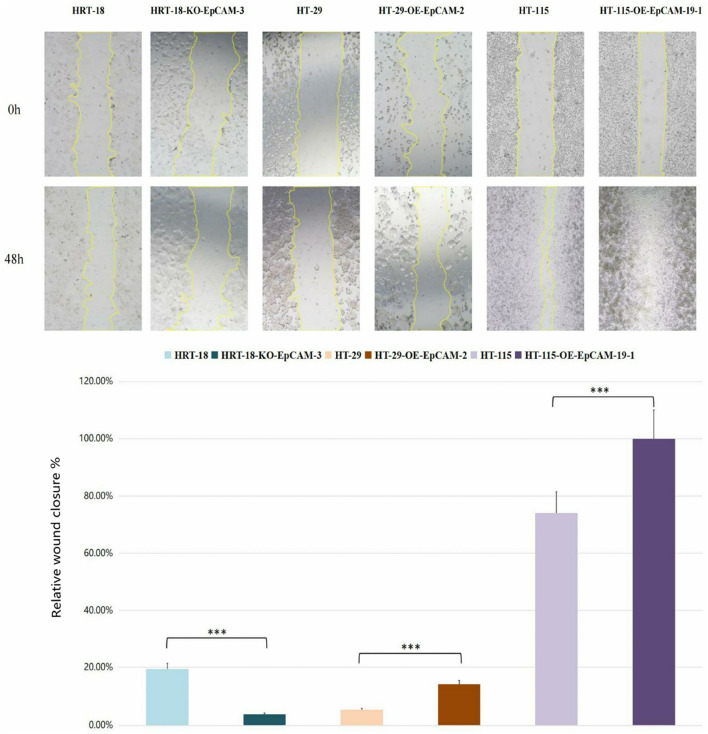
Scratch assay phenotypes at 48 h (×40 magnification).

Phenotypic hierarchy: HT-115-OE > HT-29-OE > HRT-18 WT > HRT-18-KO (Group 3 vs. Group 1: *p* < 0.001; Group 3 vs. Group 2: *p* < 0.001) (Figure 7; Table 4).

**TABLE 4:** Scratch assay quantification (48 h).

Group	Cell line	Mean (%)	SD (%)	Within-group comparison (p-value)	Between-group comparison (p-value)	Significance
Group1	HRT-18	19.45	1.59	-	-	-
	HRT-18-KO-EpCAM-3	3.79	3.36	<0.001***	-	***
	**Group1 Average**	**11.62**	**8.89**	-	vs. Group2: 0.9502	-
Group2	HT-29	5.37	3.8	-	-	-
	HT-29-OE-EpCAM-2	14.08	9.15	0.023*	-	*
	**Group2 Average**	**9.72**	**7.87**	-	vs. Group3: <0.001***	***
Group3	HT-115	74.05	3.58	-	-	-
	HT-115-OE-EpCAM-19–1	100	0	<0.001***	-	***
	**Group3 Average**	**87.02**	**14.39**	-	vs. Group1: <0.001***	***

#### 3.8.2 Transwell chemotaxis

Significant inter-group variation was observed (ANOVA: *F* = 7.13, *p* = 0.0024).

Group 2 (HT-29 series): Highest migration (*p* = 0.0021 vs. Group 1)

Group 3 (HT-115 series): Intermediate phenotype (*p* = 0.0415 vs. Group 2).

**FIGURE 8:**
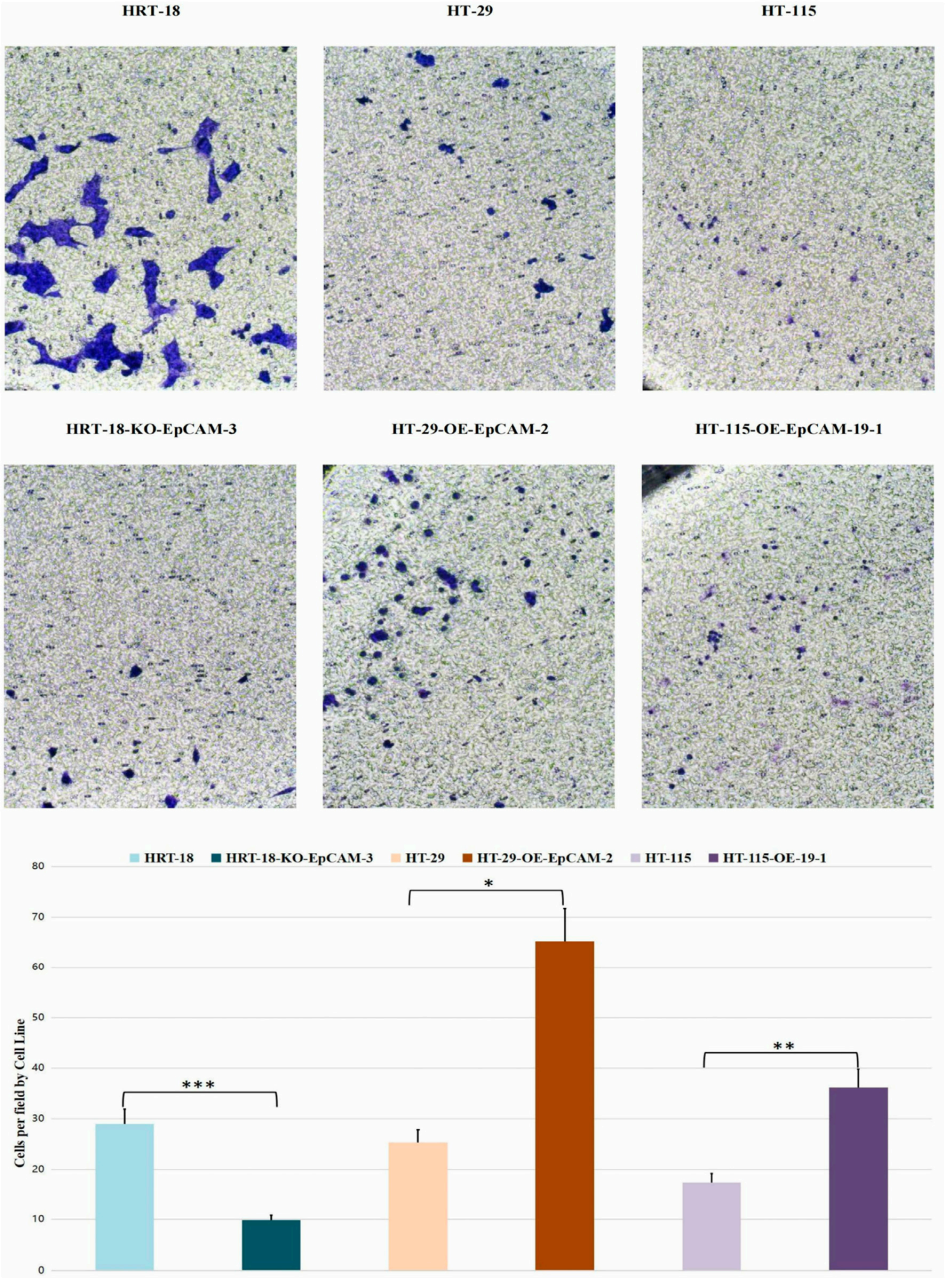
Transwell assays.

Group 1 (HRT-18 series): Lowest capacity (*p* = 0.5754 vs. Group 3, NS) (Figure 8).

### 3.9 Integrated phenotyping informs translational utility

Comprehensive functional mapping established model-specific applications:

HT-29-OE-EpCAM-2: Combines maximal proliferation↑ (20.1%), collective migration↑ (2.6-fold), and peak chemotactic response–optimal for metastatic drug screening.

HT-115-OE-EpCAM-19: Exhibits barrier-saturating scratch closure (100%) despite moderate proliferation↑ (11.5%) – ideal for tissue penetration studies.

HRT-18-KO-EpCAM-3: Shows profound migration↓↓ (80.5%) with minimal proliferation↓ (9.7%) – validated platform for therapeutic safety assessment (Table 5).

**TABLE 5:** Integrated phenotypic profiling reveals clinical implications.

Parameter	HRT-18-KO	HT-29-OE	HT-115-OE
Proliferation	↓ (9.7%)	↑↑↑ (20.1%)	↑ (11.5%)
Scratch	↓↓↓ (80.5%)	↑↑ (2.6-fold)	↑↑↑ (100%)
Transwell	↓ (Lowest)	↑↑↑ (Highest)	↑ (Intermediate)
Clinical Utility	Safety assessment	Metastasis drug screening	Barrier-crossing studies

## 4 Discussion

Colorectal cancer is a highly prevalent digestive system malignancy worldwide, with both high incidence and mortality rates. The increasing life expectancy and the aging population in our country contribute to the rising rates of colorectal cancer. Currently, the main treatments for colorectal cancer are surgery, radiation therapy, chemotherapy, targeted therapies, and neoadjuvant therapies. Surgical treatment is primarily effective for early-stage colorectal cancer; integrating radiotherapy and chemotherapy can prevent recurrence and metastasis. For advanced colorectal cancer patients, surgical treatment often serves a palliative function.

In recent years, rapid advancements in targeted therapies and immunotherapy have provided new hope for treating advanced and metastatic colorectal cancer. Whether through monoclonal antibody treatments targeting specific tumor molecules or through immunotherapies using immune checkpoint inhibitors or genetically modified immune cells, selecting appropriate therapeutic targets is essential for effectiveness.

Epithelial Cell Adhesion Molecule (EpCAM) is a type I transmembrane glycoprotein, approximately 40 kDa in size, expressed in epithelial and epithelial-derived cancers including lung, gastric, colorectal, and other forms of epithelial tumors (Zhu et al., 2021; Zhao et al., 2023). Previous studies indicate that EpCAM is expressed at high levels in tumor stem cells and circulating tumor cells (CTCs) (Žagar et al., 2021; Brown et al., 2021), regulating intracellular signaling for cell proliferation, and modulating EMT (Chen et al., 2020; Shi et al., 2021; Wang et al., 2018); thus, it is closely associated with tumor recurrence and metastasis (Panda et al., 2024; Shi et al., 2020). Currently, EpCAM has been FDA-approved as a diagnostic marker for breast, prostate, and colorectal cancers (Lin et al., 2021), and anti-EpCAM monoclonal antibodies have been approved in Europe for treating malignant ascites in EpCAM-positive cancer patients (Eyvazi et al., 2018).

In this study, we utilized flow cytometry to evaluate the expression levels of the EpCAM gene across seven colorectal cancer cell lines, revealing inconsistent expression levels. High-expressing lines such as COLO205 and HRT-18 showed detection rates above 80%, while lines such as LOVO, CaCO2, and HT-29 demonstrated rates around 30%. Additionally, the HT-115 cell line was found to lack EpCAM expression entirely. These findings indicate the variability in expression levels, demonstrating that while EpCAM is predominantly expressed in colorectal cancer cell lines, it cannot serve as a standalone diagnostic criterion. Therefore, combining EpCAM assessment with other diagnostic factors is crucial for diagnosing colorectal cancer effectively.

Through genetic engineering techniques, we constructed one eukaryotic overexpression vector and three CRISPR/Cas9 knockdown vectors targeting the EpCAM gene, confirming successful construction via restrictively analyzing and sequencing. Based on the flow cytometry data regarding EpCAM expression across colorectal cancer cell lines, and considering the practical challenge posed by COLO205s semi-adherent properties, HT-115 and HT-29 were chosen for screening EpCAM overexpression, while HRT-18 and T84 were selected for EpCAM knockout/downregulation studies. Our results yielded successful development of EpCAM-overexpressing colorectal cancer cell lines, HT-29-OE-EpCAM-2 (73.63%) and HT-115-OE-EpCAM-19 (24.06%). Future studies will leverage flow cytometric sorting to purify high-expression EpCAM cell lines.

Regarding the selection of cell lines for EpCAM knockout or downregulation, the absence of suitable lines among the 17 monoclonal T84 cells indicates a possible structural mismatch or acquired drug resistance over extended exposure to puromycin. For HRT-18 colorectal cancer cell lines, the PGMC-KO-EpCAM-3 knockdown vector emerged as the most effective, as its sgRNA sequences targeted the first and third exons of the EpCAM gene, thus facilitating more efficient knockout or downregulation.

## 5 Summary of key findings

This study employed genetic engineering approaches to construct EpCAM overexpression and CRISPR/Cas9 knockdown vectors, establishing stable transgenic models across multiple colorectal cancer (CRC) cell lines. Functional analyses demonstrated that EpCAM expression levels directly regulate malignant phenotypes (Table 6): overexpression significantly enhanced proliferation (HT-29-OE-EpCAM-2: ↑20.1%, *p* < 0.001) and migration (scratch closure: ↑2.6-fold, *p* = 0.023; Transwell migration: highest cell count), while knockdown severely impaired migration (HRT-18-KO-EpCAM-3: ↓↓80.5%, *p* < 0.001). Notably, cell line-dependent responses were observed: metastatic HT-29 cells exhibited maximal sensitivity to EpCAM overexpression, while HRT-18 migration was highly EpCAM-dependent. This context-specificity implies microenvironmental modulation via epigenetic or transcriptional networks.

**TABLE 6:** Functional validation of EpCAM in CRC models.

Functional profile	HT-29-OE-EpCAM-2	HT-115-OE-EpCAM-19	HRT-18-KO-EpCAM-3
Proliferation	Peak density ↑20.1%*	Saturation density ↑11.5%*	Doubling time ↑8.8%*
Scratch Migration	Closure rate ↑2.6-fold*	100% wound closure*	Closure rate ↓↓80.5%*
Transwell Migration	Highest chemotaxis	Intermediate capacity	Lowest capacity
Molecular Mechanism	Wnt/β-catenin activation	Complete EMT induction	Loss of cell polarity

In particular, our functional analyses revealed that even a partial reduction of EpCAM expression (approximately 60% in the HRT-18-KD model) was sufficient to elicit a significant impairment in migratory capacity (a decrease of over 80%). This pronounced phenotypic effect underscores the critical role EpCAM plays in driving the aggressive behavior of this particular cell line and suggests that its function may be particularly susceptible to dosage reduction.

It is important to note that while our novel CRISPR/Cas9 strategy targeting exons 1 and 3 successfully achieved a significant knockdown, achieving a complete genetic knockout of EpCAM remains a considerable technical challenge. Potential factors contributing to this challenge include the high efficiency required for simultaneous editing of two alleles and the possible selection pressure against clones that completely lose this potentially vital oncoprotein. Therefore, the generation of a complete EpCAM knockout model is a recognized priority for future research, as it would allow for an even more definitive investigation of its biological functions.

Nevertheless, the robust functional consequences observed from our partial knockdown approach not only validate the effectiveness of our targeting strategy but also provide compelling evidence for EpCAM as a master regulator of CRC progression.

### 5.1 Molecular mechanisms and clinical correlations

While our *in vitro* findings provide mechanistic insights, we acknowledge the inherent limitations of cell-based models in fully recapitulating the complexity of the tumor microenvironment *in vivo*. Therefore, conclusions regarding molecular mechanisms and clinical relevance should be interpreted as preliminary and indicative. EpCAM, a multifunctional oncofetal antigen, has been implicated in aggressive traits in colorectal cancer (CRC). Jiang et al. (2023) suggested in their review that the EpCAM–β-catenin complex derepresses TCF/LEF transcription, thereby upregulating pro-proliferative genes (e.g., c-Myc, Cyclin D1 (16). Consistent with a potential oncogenic role, EpCAM expression levels have been shown to correlate with advanced Dukes staging (C + D vs. A + B; *p* < 0.05), Lymph node metastasis (positive vs. negative; *p* < 0.05), and Distant metastasis (present vs. absent; *p* < 0.05) (Wang, 2021). These collective findings position EpCAM as a promising molecular driver of CRC progression, a notion that requires further *in vivo* validation.

Our *in vitro* data suggest a potential immunoregulatory role for EpCAM, as HRT-18-KO-EpCAM-3 cells showed enhanced CD8^+^ T-cell migrationin transwell assays. This observation complements the *in vivo* findings of Kanabori et al. (Kanahori et al., 2024), who reported that EpCAMhi sarcoma lung metastases suppress CD8^+^ T-cell infiltration, while knockout restores T-cell influx. Notably, Du et al. (Wang S. et al., 2025) identified an EPCAM c.661A>G mutation driving “immune-cold” phenotypes in Lynch syndrome-associated CRC, lending additional support to the concept of EpCAM as a potential immune microenvironmental target. The proposed immunomodulatory function, potentially involving the EpICD domain regulating IL-6/STAT3 signaling, remains to be robustly validated in more complex physiological settings.

### 5.2 Translational implications

The EpCAM-engineered models provide optimized platforms for drug screening (Table 7). HT-29-OE-EpCAM-2—with rapid proliferation (doubling time: 22.4 h) and metastatic traits—is ideal for metastasis-targeted therapy. Conversely, HT-115-OE-EpCAM-19’s 100% scratch closure offers a unique model for barrier-penetrating drug evaluation (Zhang, 2016). EpCAM-targeted approaches include.

1. Antibody/aptamer-guided delivery: pH-sensitive carriers exploiting tumor acidity could refine targeting.
2. Small-molecule inhibitors: Arenobufagin docks effectively with EpCAM; HRT-18-KO enables critical off-target toxicity assessment.
3. Immunotherapy combinations: Given EpCAM knockout enhances CD8^+^ T-cell infiltration, combining anti-EpCAM antibodies with PD-1/CTLA-4 inhibitors may reverse checkpoint inhibitor resistance, especially in MSI-H CRC (Wang S. et al., 2025).

**TABLE 7:** Therapeutic development strategies based on EpCAM-Engineered models.

Application	Optimal model	Strategy	Clinical potential
Metastasis-targeted therapy	HT-29-OE-EpCAM-2	EpCAM-directed ADCs	High (micrometastasis suppression)
Immunotherapy sensitization	HRT-18-KO-EpCAM-3	Anti-EpCAM + PD-1 blockade	High (“cold” tumor reversal)
Drug delivery systems	HT-115-OE-EpCAM-19	Aptamer-decorated nanoparticles	Medium (tumor accumulation)
Small-molecule inhibitors	Tri-model parallel	Structure-guided arenobufagin optimization	High (oral bioavailability)

## 6 Limitations and future perspectives

Despite systematic characterization, limitations exist that provide avenues for future research.

### 6.1 Technical and model limitations

1. Model systems: Monolayer cultures inadequately recapitulate *in vivo* microenvironments. Spatial transcriptomics implicates fibroblast interactions via collagen/FN1 in CRC metastasis (Wang S. et al., 2025). Future work should employ patient-derived organoids (PDOs) and humanized mice integrated with single-cell sequencing to validate findings in a more physiological context.
2. Cell line selection rationale: The selection of the HT-115 cell line for overexpression studies was based on its negligible baseline EpCAM expression, which provided an ideal null background to unequivocally test the sufficiency of EpCAM. While this model was optimal for addressing this specific question, it does not recapitulate the scenario of augmenting EpCAM expression in cells with pre-existing moderate levels. Future studies employing well-characterized models like HCT-116 or SW480 would provide valuable complementary insights into the role of EpCAM in augmenting tumorigenicity.
3. Fluorescent reporter artifact: A noticeable discrepancy between EpCAM and RFP expression levels was observed due to the nature of the multicistronic vector (T2A-linked EpCAM-RFP-PuroR cassette). The RFP signal should be interpreted as a qualitative marker of transduction, not a quantitative one, with overexpression definitively assessed by target-specific antibodies.

### 6.2 Molecular and functional characterization gaps

1. Molecular networks: EpCAM–ncRNA interactions (e.g., lncRNA-TINCR regulating EpCAM proteolysis (Jiang et al., 2023)) and impact on key signaling pathways (e.g., Wnt/β-catenin, PI3K/Akt) and EMT markers remain unexplored. Deciphering these molecular drivers is a crucial next step.
2. Incomplete mechanistic validation: While our novel CRISPR/Cas9 strategy targeting exons 1 and 3 of EpCAM is designed to prevent compensatory alternative splicing—a known pitfall of conventional exon 2 targeting (Bagheri et al., 2022; Wang J. et al., 2025; Sun et al., 2025)—and strong functional impairment was observed, future studies should directly sequence the edited transcripts to conclusively confirm the absence of escape variants.
3. Phenotypic assay scope: We focused on 2D migration models. The inclusion of 3D invasion assays (e.g., using Matrigel-coated inserts (Justus et al., 2014)) would provide a more physiologically relevant assessment of invasive potential.

### 6.3 Translational and therapeutic challenges

1. Therapeutic window: Basal EpCAM expression in normal epithelia risks on-target toxicity, necessitating the development of conditionally activated antibodies or bispecifics (e.g., EpCAM × CD3 (Yao et al., 2014)) to improve specificity.
2. *In vivo* relevance: The *in vivo* relevance of our findings requires validation in animal models, such as xenograft studies, to confirm the role of EpCAM in tumor growth and metastasis within a complex tumor microenvironment (Frese and Tuveson, 2007).

### 6.4 Future research and clinical perspectives

Building on this work, which establishes genetically engineered EpCAM models defining its pivotal role in CRC proliferation and metastasis, future efforts should prioritize.

1. Next-generation EpCAM targeting: Develop AI-designed allosteric inhibitors or bispecific antibodies to minimize off-target effects.
2. Combination therapies: Explore synergistic approaches with immune checkpoint or epigenetic inhibitors (e.g., anti-EpCAM + PD-1 blockade in MSI-H CRC).
3. Liquid biopsy applications: Exploit EpCAM for CTC capture coupled with single-cell sequencing to predict early recurrence.
4. Technology application: Our exon 1/3 targeting strategy provides a robust framework for complete gene disruption that could be applicable to other therapeutic targets prone to alternative splicing.

In summary, overcoming these limitations and exploring these future directions will be essential to fully exploit EpCAM’s diagnostic, prognostic, and therapeutic versatility and advance CRC precision medicine.

## Data Availability

The raw data supporting the conclusions of this article will be made available by the authors, without undue reservation.

## References

[B1] Agnoletto C. Caruso C. Garofalo C. (2021). Heterogeneous circulating tumor cells in sarcoma: implication for clinical practice. Cancers (Basel) 13 (9), 2189. 10.3390/cancers13092189 34063272 PMC8124844

[B2] Bagheri A. Culp P. A. DuBridge R. B. Chen T. T. (2022). CRISPR/Cas9 disruption of EpCAM exon 2 results in cell-surface expression of a truncated protein targeted by an EpCAM specific T cell engager. Biochem. Biophys. Rep. 29, 101205. 10.1016/j.bbrep.2022.101205 35071801 PMC8761601

[B3] Balzar M. Bakker H. A. Briaire-de-Bruijn I. H. Fleuren G. J. Warnaar S. O. Litvinov S. V. (1998). Cytoplasmic tail regulates the intercellular adhesion function of the epithelial cell adhesion molecule. Mol. Cell Biol. 18 (8), 4833–4843. 10.1128/MCB.18.8.4833 9671492 PMC109068

[B4] Brown T. C. Sankpal N. V. Gillanders W. E. (2021). Functional implications of the dynamic regulation of EpCAM during epithelial-to-mesenchymal transition. Biomolecules 11 (7), 956. 10.3390/biom11070956 34209658 PMC8301972

[B5] Chen H. N. Liang K. H. Lai J. K. Lan C. H. Liao M. Y. Hung S. H. (2020). EpCAM signaling promotes tumor progression and protein stability of PD-L1 through the EGFR pathway. Cancer Res. 80 (22), 5035–5050. 10.1158/0008-5472.CAN-20-1264 32978170

[B6] Eyvazi S. Farajnia S. Dastmalchi S. Kanipour F. Zarredar H. Bandehpour M. (2018). Antibody based EpCAM targeted therapy of cancer, review and update. Curr. Cancer Drug Targets 18 (9), 857–868. 10.2174/1568009618666180102102311 29295696

[B7] Ezenkwa U. S. Ogun G. O. Mashor M. I. Ogunbiyi O. J. (2023). EpCAM expression negatively regulates E-cadherin function in colorectal carcinomas. Ecancermedicalscience 17, 1569. 10.3332/ecancer.2023.1569 37533952 PMC10393316

[B8] Frese K. K. Tuveson D. A. (2007). Maximizing mouse cancer models. Nat. Rev. Cancer 7 (9), 645–658. 10.1038/nrc2192 17687385

[B9] Gaber A. Lenarčič B. Pavšič M. (2020). Current view on EpCAM structural biology. Cells 9 (6), 1361. 10.3390/cells9061361 32486423 PMC7349879

[B10] Gires O. Pan M. Schinke H. Canis M. Baeuerle P. A. (2020). Expression and function of epithelial cell adhesion molecule EpCAM: where are we after 40 years? Cancer Metastasis Rev. 39 (3), 969–987. 10.1007/s10555-020-09898-3 32507912 PMC7497325

[B11] Hosono H. Ohishi T. Takei J. Asano T. Sayama Y. Kawada M. (2020). The anti-epithelial cell adhesion molecule (EpCAM) monoclonal antibody EpMab-16 exerts antitumor activity in a mouse model of colorectal adenocarcinoma. Oncol. Lett. 20 (6), 383. 10.3892/ol.2020.12246 33154781 PMC7608076

[B12] Jiang X. Wang S. Liang Q. Liu Y. Liu L. (2023). Unraveling the multifaceted role of EpCAM in colorectal cancer: an integrated review of its function and interplay with non-coding RNAs. Med. Oncol. 41 (1), 35. 10.1007/s12032-023-02273-6 38151631

[B13] Justus C. R. Leffler N. Ruiz-Echevarria M. Yang L. V. (2014). *In vitro* cell migration and invasion assays. J. Vis. Exp. (88), 51046. 10.3791/51046 24962652 PMC4186330

[B14] Kanahori M. Shimada E. Matsumoto Y. Endo M. Fujiwara T. Nabeshima A. (2024). Immune evasion in lung metastasis of leiomyosarcoma: upregulation of EPCAM inhibits CD8^+^ T cell infiltration. Br. J. Cancer 130 (7), 1083–1095. 10.1038/s41416-024-02576-z 38291183 PMC10991329

[B15] Keller L. Werner S. Pantel K. (2019). Biology and clinical relevance of EpCAM. Cell Stress 3 (6), 165–180. 10.15698/cst2019.06.188 31225512 PMC6558934

[B16] Lee C. C. Yu C. J. Panda S. S. Chen K. C. Liang K. H. Huang W. C. (2024). Correction: epithelial cell adhesion molecule (EpCAM) regulates HGFR signaling to promote colon cancer progression and metastasis. J. Transl. Med. 22 (1), 36. 10.1186/s12967-023-04797-x 38191443 PMC10775552

[B17] Lin D. Shen L. Luo M. Zhang K. Li J. Yang Q. (2021). Circulating tumor cells: biology and clinical significance. Signal Transduct. Target Ther. 6 (1), 404. 10.1038/s41392-021-00817-8 34803167 PMC8606574

[B18] Macdonald J. Henri J. Roy K. Hays E. Bauer M. Veedu R. N. (2018). EpCAM immunotherapy versus specific targeted delivery of drugs. Cancers (Basel) 10 (1), 19. 10.3390/cancers10010019 29329202 PMC5789369

[B19] Mederer T. Elsner F. Robold T. Großer C. Neu R. Ried M. (2022). EpCAM-positive disseminated cancer cells in bone marrow impact on survival of early-stage NSCLC patients. Lung Cancer 167, 73–77. 10.1016/j.lungcan.2022.02.008 35421717

[B20] Menyailo M. E. Bokova U. A. Ivanyuk E. E. Khozyainova A. A. Denisov E. V. (2021). Metastasis prevention: focus on metastatic circulating tumor cells. Mol. Diagn Ther. 25 (5), 549–562. 10.1007/s40291-021-00543-5 34287797

[B21] Mirzaei R. Shafiee S. Vafaei R. Salehi M. Jalili N. Nazerian Z. (2023). Production of novel recombinant anti-EpCAM antibody as targeted therapy for breast cancer. Int. Immunopharmacol. 122, 110656. 10.1016/j.intimp.2023.110656 37473710

[B22] Panda S. S. Lee C. C. Geevimaan K. Chen K. C. Yang S. H. Shen C. N. (2024). Intracellular domain of epithelial cell adhesion molecule induces wnt receptor transcription to promote colorectal cancer progression. J. Biomed. Sci. 31 (1), 72. 10.1186/s12929-024-01057-y 39010070 PMC11247908

[B23] Patriarca C. Macchi R. M. Marschner A. K. Mellstedt H. (2012). Epithelial cell adhesion molecule expression (CD326) in cancer: a short review. Cancer Treat. Rev. 38 (1), 68–75. 10.1016/j.ctrv.2011.04.002 21576002

[B24] Schmidt M. Rüttinger D. Sebastian M. Hanusch C. A. Marschner N. Baeuerle P. A. (2012). Phase IB study of the EpCAM antibody adecatumumab combined with docetaxel in patients with EpCAM-positive relapsed or refractory advanced-stage breast cancer. Ann. Oncol. 23 (9), 2306–2313. 10.1093/annonc/mdr625 22357251

[B25] Shi R. Liu L. Wang F. He Y. Niu Y. Wang C. (2020). Downregulation of cytokeratin 18 induces cellular partial EMT and stemness through increasing EpCAM expression in breast cancer. Cell Signal 76, 109810. 10.1016/j.cellsig.2020.109810 33069797

[B26] Shi R. Z. He Y. F. Wen J. Niu Y. N. Gao Y. Liu L. H. (2021). Epithelial cell adhesion molecule promotes breast cancer resistance protein-mediated multidrug resistance in breast cancer by inducing partial epithelial-mesenchymal transition. Cell Biol. Int. 45 (8), 1644–1653. 10.1002/cbin.11598 33760350

[B27] Slanchev K. Carney T. J. Stemmler M. P. Koschorz B. Amsterdam A. Schwarz H. (2009). The epithelial cell adhesion molecule EpCAM is required for epithelial morphogenesis and integrity during zebrafish epiboly and skin development. PLoS Genet. 5 (7), e1000563. 10.1371/journal.pgen.1000563 19609345 PMC2700972

[B28] Sun Q. Ma X. Ning Q. Li S. Wang P. Tan X. (2025). Systematic screening for functional exon-skipping isoforms using the CRISPR-RfxCas13d system. Cell Syst. 16 (8), 101351. 10.1016/j.cels.2025.101351 40763746

[B29] Treitschke S. Weidele K. Varadarajan A. R. Feliciello G. Warfsmann J. Vorbeck S. (2023). *Ex vivo* expansion of lung cancer-derived disseminated cancer cells from lymph nodes identifies cells associated with metastatic progression. Int. J. Cancer 153 (10), 1854–1867. 10.1002/ijc.34658 37555668

[B30] Tsaktanis T. Kremling H. Pavšič M. von Stackelberg R. Mack B. Fukumori A. (2016). Cleavage and cell adhesion properties of human epithelial cell adhesion molecule (HEPCAM). J. Biol. Chem. 291 (1), 425. 10.1074/jbc.A115.662700 26724312 PMC4697179

[B31] Wang L. (2021). Correlation between EpCAM expression and clinicopathological features and diagnostic accuracy in colorectal cancer: a meta-analysis. Master’s thesis. Lanzhou, China: Lanzhou University.

[B32] Wang M. H. Sun R. Zhou X. M. Zhang M. Y. Lu J. B. Yang Y. (2018). Epithelial cell adhesion molecule overexpression regulates epithelial-mesenchymal transition, stemness and metastasis of nasopharyngeal carcinoma cells via the PTEN/AKT/mTOR pathway. Cell Death Dis. 9 (1), 2. 10.1038/s41419-017-0013-8 29305578 PMC5849035

[B33] Wang S. Zhang K. Cheng Y. Liu L. Du M. (2025). A EPCAM pathogenic variant in familial lynch syndrome-associated Colon cancer: insights into genetic basis and tumor microenvironment characteristics. Phenomics 5, 183–191. 10.1007/s43657-024-00202-9 40606558 PMC12209482

[B34] Wang J. Wang F. Huang H. (2025). EXO1 as a potential biomarker for prognosis, immune infiltration, and immunotherapy in pan-cancer analysis. Funct. Integr. Genomics 25 (1), 87. 10.1007/s10142-025-01586-1 40214853

[B35] Winter M. J. Nagelkerken B. Mertens A. E. Rees-Bakker H. A. Briaire-de Bruijn I. H. Litvinov S. V. (2003). Expression of Ep-CAM shifts the state of cadherin-mediated adhesions from strong to weak. Exp. Cell Res. 285 (1), 50–58. 10.1016/s0014-4827(02)00045-9 12681286

[B36] Xu T. Schulga A. Konovalova E. Rinne S. S. Zhang H. Vorontsova O. (2023). Feasibility of Co-Targeting HER3 and EpCAM using seribantumab and DARPin-Toxin fusion in a pancreatic cancer xenograft model. Int. J. Mol. Sci. 24 (3), 2838. 10.3390/ijms24032838 36769161 PMC9917732

[B37] Yao X. Williamson C. Adalsteinsson V. A. D'Agostino R. S. Fitton T. Smaroff G. G. (2014). Tumor cells are dislodged into the pulmonary vein during lobectomy. J. Thorac. Cardiovasc Surg. 148 (6), 3224–31.e315. 10.1016/j.jtcvs.2014.06.074 25172322 PMC4356533

[B38] Žagar T. Pavšič M. Gaber A. (2021). Destabilization of EpCAM dimer is associated with increased susceptibility towards cleavage by TACE. PeerJ 9, e11484. 10.7717/peerj.11484 34055495 PMC8142927

[B39] Zhang Z. Y. (2016). Deficiency of long non-coding RNA-TINCR regulates EpCAM proteolytic cleavage to promote proliferation and metastasis in colorectal cancer. Doctoral dissertation. Guangzhou, China: Southern Medical University.

[B40] Zhao X. Zhao R. Feng Y. Qiu Z. Bai X. Zhang D. (2023). The roles EpCAM plays to enhance the malignancy of gastric cancer. J. Cancer Res. Clin. Oncol. 149 (11), 8495–8505. 10.1007/s00432-023-04767-2 37095412 PMC11797087

[B41] Zhu T. Peng X. Cheng Z. Xing D. Zhang M. (2021). Diagnostic rather than prognostic markers-relationship between EpCAM overexpression and lung cancer: a meta-analysis. Ann. Palliat. Med. 10 (4), 4025–4036. 10.21037/apm-20-2013 33832309

